# Supplementary material for: Splice-Junction-Based Mapping of Alternative Isoforms in the Human Proteome
Source: Cell Rep. Author manuscript; Available in PMC 2020 Jan 15. (PMC6961840; doi:10.1016/j.celrep.2019.11.026)
Supplement: 2 [file NIHMS1546469-supplement-2.zip › DF1/PXD006675/SmoothMuscleCells/SmoothMuscleCells_11_CD46_FTSITPFPSWR.pdf]

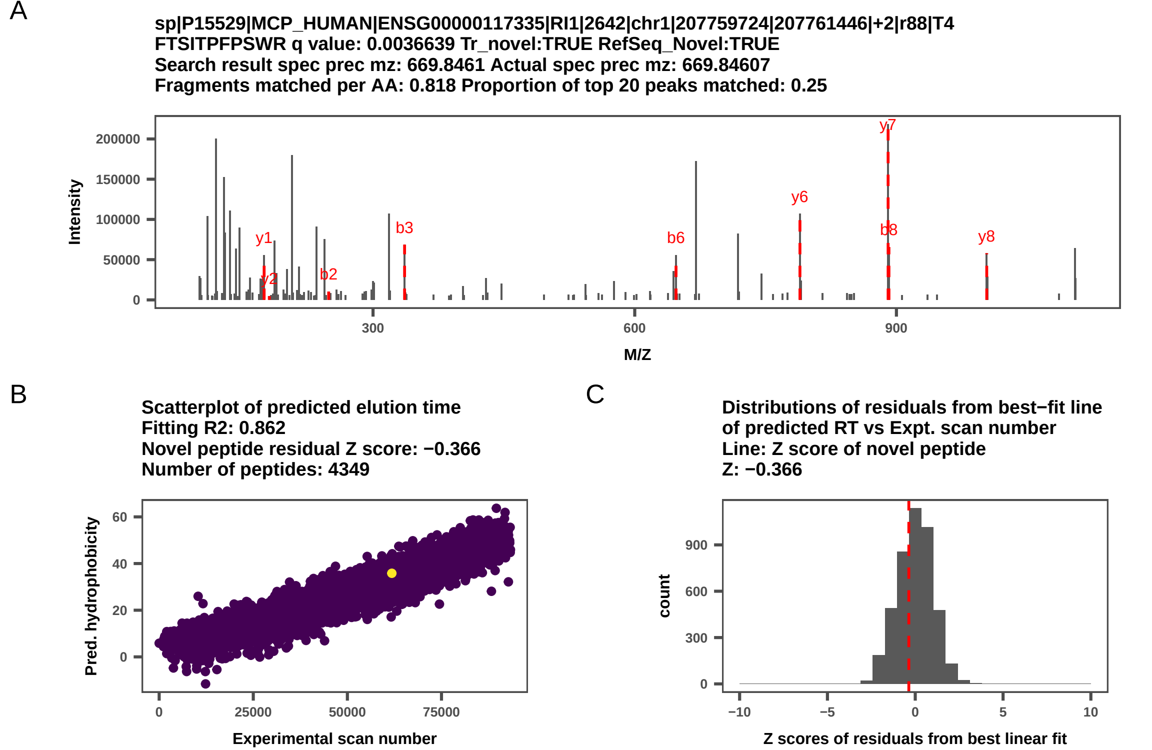
A
sp|P15529|MCP_HUMAN|ENSG00000117335|RI1|2642|chr1|207759724|207761446|+2|r88|T4
FTSITPFPSWR q value: 0.0036639 Tr_novel:TRUE RefSeq_Novel:TRUE
Search result spec prec mz: 669.8461 Actual spec prec mz: 669.84607
Fragments matched per AA: 0.818 Proportion of top 20 peaks matched: 0.25
Intensity
200000
150000
100000
50000
0
y1
y2
b2
b3
b6
y6
y7
b8
y8
300
600
900
M/Z
B
Scatterplot of predicted elution time
Fitting R2: 0.862
Novel peptide residual Z score: −0.366
Number of peptides: 4349
Pred. hydrophobicity
60
40
20
0
0
25000
50000
75000
Experimental scan number
C
Distributions of residuals from best−fit line
of predicted RT vs Expt. scan number
Line: Z score of novel peptide
Z: −0.366
count
900
600
300
0
−10
−5
0
5
10
Z scores of residuals from best linear fit
